# Cognitive and linguistic abilities and perceptual restoration of missing speech: Evidence from online assessment

**DOI:** 10.3389/fpsyg.2022.1059192

**Published:** 2022-12-08

**Authors:** Andrew M. Burleson, Pamela E. Souza

**Affiliations:** Hearing Aid Laboratory, Department of Communication Sciences and Disorders, Northwestern University, Evanston, IL, United States

**Keywords:** perceptual restoration, interrupted speech, cognition, linguistic, online assessment

## Abstract

When speech is clear, speech understanding is a relatively simple and automatic process. However, when the acoustic signal is degraded, top-down cognitive and linguistic abilities, such as working memory capacity, lexical knowledge (i.e., vocabulary), inhibitory control, and processing speed can often support speech understanding. This study examined whether listeners aged 22–63 (mean age 42 years) with better cognitive and linguistic abilities would be better able to perceptually restore missing speech information than those with poorer scores. Additionally, the role of context and everyday speech was investigated using high-context, low-context, and realistic speech corpi to explore these effects. Sixty-three adult participants with self-reported normal hearing completed a short cognitive and linguistic battery before listening to sentences interrupted by silent gaps or noise bursts. Results indicated that working memory was the most reliable predictor of perceptual restoration ability, followed by lexical knowledge, and inhibitory control and processing speed. Generally, silent gap conditions were related to and predicted by a broader range of cognitive abilities, whereas noise burst conditions were related to working memory capacity and inhibitory control. These findings suggest that higher-order cognitive and linguistic abilities facilitate the top-down restoration of missing speech information and contribute to individual variability in perceptual restoration.

## Introduction

When conditions are optimal, understanding speech for normal-hearing listeners is a relatively simple and automatic process. High-fidelity speech information is rapidly transmitted through the peripheral and central auditory systems to the primary auditory cortex, acoustic cues are matched to stored lexical representations, and meaning can be extracted with little to no conscious reappraisal (Marslen-Wilson and Welsh, 1978). However, in everyday communication, background noise or interruptions are common. Background noise can interfere with the perception of target speech (Gordon-Salant and Fitzgibbons, 2004; Neuman et al., 2010; Smith et al., 2019), and if the interruptions become more intense than the speech itself, segments may be masked entirely. Despite this obfuscation of the speech signal by background noise, many listeners with normal hearing remain able to understand speech relatively well. In difficult conditions, listeners are thought to piece together the remaining speech fragments to “fill in the gaps,” integrating and organizing them perceptually across time. For example, listeners may rely on spectrotemporal cues, such as the fundamental frequency, that are less affected by background noise and can bridge the gaps to assist with perceptual grouping (Li and Loizou, 2007; Oxenham, 2008). This idea is often referred to as “glimpsing,” “dip listening” (Cooke, 2006; Akeroyd, 2008), or “perceptual restoration” when segments of speech information are intentionally absent or removed (Warren, 1970).

This process can be investigated using an interrupted speech paradigm wherein segments of a speech signal are periodically removed, as pioneered by Miller and Licklider (1950). Periodic removal of speech allows for an investigation into which factors may aid in the recovery of the remaining proportion of speech information. Speech intelligibility improves when the periodic interruption is a rectangular burst of broadband noise instead of a silent gap (Powers and Wilcox, 1977; Bashford and Warren, 1987; Bashford et al., 1992; Bologna et al., 2019). This effect is particularly salient when the intensity of the noise burst is greater than the speech signal. The negative signal-to-noise ratio is thought to give the listener the impression of perceptually continuous speech occurring behind the noise, aiding perceptual organization and grouping (Bashford and Warren, 1987; Bashford et al., 1996).

Most perceptual restoration research has emphasized signal-level factors, such as speech spectrotemporal fidelity, rate, and length; interruption length, density, and type; and interruption/signal intensity that affect how much speech information can be restored (Miller and Licklider, 1950; Warren, 1970; Bashford et al., 1996; Başkent et al., 2009; Chatterjee et al., 2010; Jin and Nelson, 2010; Benard et al., 2014; Clarke et al., 2016; Shafiro et al., 2016). Furthermore, most of the perceptual restoration literature focuses on group differences (e.g., age, hearing status) (Başkent, 2010; Kidd and Humes, 2012; Fogerty et al., 2015; Başkent et al., 2016; Bologna et al., 2018; Jaekel et al., 2018). While signal-level factors clearly play a role in the ability to restore missing speech, individuals within the same group still vary substantially in their perceptual restoration ability.

We suggest that individual variability during the perceptual restoration of missing speech information may be driven by individual differences in higher-order processing abilities, such as cognitive and linguistic abilities. Cognitive abilities differ substantially from one person to the next, and while abilities such as working memory and inhibitory control do tend to vary together within one person, they are not always aligned (e.g., an individual can have high working memory with low inhibitory control) (Carroll and Maxwell, 1979; Boogert et al., 2018). Linguistic abilities also vary across individuals, with lexical knowledge, or vocabulary, increasing with advancing age (Verhaeghen, 2003). The Ease of Language Understanding (ELU) model takes these higher-order processes into account, proposing a model where cognitive and linguistic abilities interact to support degraded speech understanding. The ELU model accounts for cognitive abilities, such as working memory, which allows speech to be temporarily held in an episodic buffer for later reprocessing; and linguistic knowledge, which allows context and vocabulary to identify possible lexical candidates for speech which was not automatically recognized (Rönnberg et al., 2013). This model provides an explanation whereby individuals may restore missing or interrupted speech differently based on their cognitive and linguistic abilities, as follows.

First, the reconstruction of missing speech requires the reprocessing of available speech fragments which are temporarily held in an episodic buffer. Temporarily holding speech fragments tasks a listener’s working memory capacity. Some evidence suggests that an individual’s working memory capacity mediates the ability to restore missing speech (Benard and Başkent, 2014; Millman and Mattys, 2017; Nagaraj and Magimairaj, 2017) while other data are less definitive (Nagaraj and Knapp, 2015; Shafiro et al., 2015; Bologna et al., 2018). Second, reprocessing is informed by a listener’s lexical knowledge and how quickly that information can be accessed to accurately identify lexical candidates when filling the gap. Current literature indicates that lexical knowledge (i.e., vocabulary) plays a role during perceptual restoration (Benard and Başkent, 2014; Nagaraj and Magimairaj, 2017). However, existing literature has not captured an aspect of speech perception that may be important during perceptual restoration: lexical access speed, or the rate at which stored lexical representations can be activated or matched by speech information. Third, irrelevant information, such as unlikely lexical candidates, noise-burst interruptions, and other cognitive processes that may be competing for attention must be inhibited, which relies on a listener’s inhibitory control. Current evidence suggests that an individual’s inhibitory control is predictive of his/her ability to perform other degraded speech recognition tasks, such as speech-in-noise (Dey and Sommers, 2015; Dryden et al., 2017; Stenbäck et al., 2021; Perron et al., 2022). Bologna et al. (2018) investigated inhibitory control using interrupted speech and found null results for both younger and older adults, albeit at a very difficult signal-to-noise ratio. Last, working memory reprocessing, lexical processing, and inhibitory control require processing time to complete. Thus, these abilities depend on processing speed, or the rate at which cognitive tasks are completed by an individual (Salthouse, 1992; Morrison and Gibbons, 2006; Rozas et al., 2008). Processing speed has shown predictive value in previous research on degraded speech recognition (Ellis et al., 2016; Dryden et al., 2017; Yumba, 2017) and perceptual restoration (Bologna et al., 2018), but data exist only for young normal-hearing and older hearing-impaired adults.

Taken together, we predict that listeners who have a higher working memory capacity, greater lexical knowledge, faster lexical access speed, better inhibitory control, and faster processing speed will be more successful when restoring missing speech information, especially for high context, predictable sentences. Building from previous work, we used a periodically interrupted speech paradigm to force listeners into an explicit processing loop as outlined in the ELU model (Rönnberg et al., 2013), and we separately measured the cognitive and linguistic processes supporting explicit processing. To further explore the role of lexical processing during perceptual restoration, we chose to include both high- and low-context sentences in addition to a sentence set that resembles everyday speech. Because in-person testing capacity was restricted as a result of the COVID-19 pandemic, this experiment was conducted using online assessments for listeners ranging from young to middle-aged adults.

## Materials and methods

### Participants

Prior to data collection an *a priori* power analysis was performed based on the relationship between perceptual restoration and cognitive data from Nagaraj and Magimairaj (2017). For a medium effect size of 0.3, an alpha level of 0.05, a power level of 0.9, and four predictors, the projected sample size necessary was 57 participants. Sixty-three participants (22 males, 36 females, five other or prefer not to answer) completed this experiment (Age range = 22–63 years, Mean = 42.0 years, SD = 12 years); they represented an age range captured in only a small set of perceptual restoration data (Millman and Mattys, 2017). To be eligible, participants needed to self-report normal hearing and cognitive status, speak English as their primary language, be between 18 and 65 years of age, and be a current resident of the United States. Because participation was virtual, hearing thresholds were not assessed. The Institutional Review Board of Northwestern University approved the study, all participants signed an informed consent form on the secure data collection platform REDCap (Harris et al., 2009), and participants were compensated at an hourly rate for taking part in the study.

### Stimuli

Speech stimuli consisted of three sentence sets: the Revised Speech in Noise (RSPIN) low- and high-context sentences (Bilger et al., 1984) and the Perceptually Robust English Sentence Test: Open Set (PRESTO) (Tamati et al., 2013). The RSPIN sentences were designed to determine the role of top-down and bottom-up processes during speech recognition and were selected from a corpus of 200 sentences that were highly predictable (e.g., “the witness took a solemn *oath*”), where top-down resources can inform final word choice, or 200 sentences that were unpredictable but syntactically correct (e.g., “he has a problem with the *oath*”) which relies more on the fidelity of the bottom-up signal to the auditory cortex. Following Jenstad and Souza (2007), the entire sentence was scored (see Section “General procedure”). RSPIN sentences were produced by a male talker. High context sentences had an average of 5.1 content words per sentence, and low context sentences had an average of 4.8 content words per sentence. The PRESTO is a high-variability sentence set designed to be sensitive to individual differences and is thought to access both central cognitive and perceptual abilities during speech recognition, including current theories of lexical organization and automatic encoding of lexical components. The PRESTO sentence set is balanced for talker gender, number of keywords (average of 4.2 content words per sentence), word frequency, and word familiarity.

Both silent gap sentences and noise burst sentences were constructed using a common method for interrupted speech stimuli development which is known to induce perceptual restoration. This method also avoids both floor and ceiling effects, as follows.

#### All sentences

Six sentence conditions consisting of sixty sentences each were tested: two interruption conditions (silent gap versus noise burst) by three sentence conditions (RSPIN low context, RSPIN high context, and PRESTO), resulting in 240 RSPIN sentences and 120 PRESTO sentences. First, the 360 sentences were gated with a 50% duty cycle square wave at a rate of 2.0 Hz using a custom MATLAB R2020a script, creating interrupted speech stimuli with alternating 250 ms segments of speech and silence. Second, a separate set of 360 noise-burst stimuli were created where the noise bursts aligned with the silent segments of the interrupted speech stimuli. The speech-shaped noise bursts were generated using the combined Fourier transform of all 360 sentences, where the phases of all spectral components were randomized before being converted back into the time domain using an inverse Fourier transform. The overall lengths of the noise-burst stimuli were the same as the overall lengths of the interrupted speech segments because the noise bursts would later be interleaved with the interrupted speech segments (i.e., creating alternating 250 ms segments of speech and noise bursts). To minimize spectral splatter and distortion, 10 ms cosine on- and off-ramps were applied to both the remaining interrupted speech segments and noise burst stimuli. The RMS of the interrupted speech stimuli and the noise burst stimuli were normalized. Because the amount of speech information restored improves with the addition of a noise burst when the noise burst is louder than the remaining speech segments (Bashford et al., 1996), the level of the noise burst stimuli was raised by 10 dB (–10 dB SNR) relative to all interrupted speech segments. The RMS of interrupted speech segments and the noise bursts was then normalized. By processing the stimuli this way, the level of the speech is always the same for both the silent-gap and noise-burst sentences, while the level of the noise burst will be 10 dB higher than the speech for the noise-burst sentences after processing.

#### Silent gap sentences

Silent gap sentences consisted of half of the original 360 sentences (120 RSPIN high- and low-context and 60 PRESTO sentences). The preceding procedure resulted in a set of silent gap interrupted speech stimuli with alternating segments of 250 ms of speech and 250 ms of silence with 10 ms cosine on- and off-ramps with a normalized RMS; no further signal processing was required.

#### Noise burst sentences

For the remaining half of the sentences (120 RSPIN high- and low-context and 60 PRESTO sentences), a periodic noise burst filled the silent gap. To do this, the interrupted speech segment stimuli and the noise-burst stimuli were added linearly to one another, including their individual 10 ms on- and off-ramps eliminating distortion and spectral splatter.

### General procedure

Testing was carried out using the online recruitment and experimental testing platforms Prolific and Gorilla, respectively. Pre-screening criteria (see Section “Participants”) was entered into Prolific to identify potential eligible participants, who, after indicating interest, were directed to REDCap (Harris et al., 2009), a secure data collection platform, to complete the consent form, enter demographic data, complete a brief hearing health questionnaire, and to complete the Speech and Spatial Qualities questionnaire (see Section “Questionnaires”). From there, participants were directed to the experimental platform, Gorilla, where they completed cognitive and linguistic tasks (see Sections “Cognitive tasks and Linguistic task”), a headphone screening task, and the interrupted speech task (see Section “Stimuli”).

### Questionnaires

First, participants completed a simple demographic questionnaire, followed by a hearing health questionnaire that included self-report of hearing loss and cognitive or memory concerns (participants were excluded if they answered “yes”). Last, participants completed the 49-item Speech and Spatial Qualities of Hearing questionnaire (SSQ) in which participants self-assessed their hearing ability in specific contexts and situations on a numerical scale of 0–10 (Gatehouse and Noble, 2004). Questions address self-perceived function in three domains: speech hearing (*“SSQ—Speech”)*, spatial hearing (*“SSQ—Spatial”*), and quality of hearing (*“SSQ—Quality”*). Participants were asked to rate their ability to hear and understand speech in different settings (speech hearing domain), their ability to listen in different environments, which includes distance, direction, and movement (spatial hearing domain), and their perceived abilities for everyday sounds, including music listening, ease of listening, clarity, and naturalness of sound (quality of hearing domain).

### Cognitive tasks

To assess listeners’ working memory capacity, inhibitory control, and processing speed, participants completed several automated, virtual assessments in the visual modality: the Reading Span Task (RST; complex working memory capacity), the Digit Span Forward and Backward (DST; simple working memory capacity), the Stroop Task (Stroop; processing speed and inhibitory control), and the Flanker Task (Flanker; processing speed and inhibitory control).

#### Reading span task

The reading span task (RST) is a task that measures a listener’s complex working memory capacity, or the simultaneous storage and reprocessing of complex information, requiring additional processing beyond simple repetition or reversal of information (see Section “Digit span forward and backward”). The current version of the RST was described by Rönnberg et al. (1989), which was modified from the original version first introduced by Daneman and Carpenter (1980). The current version was modified so that the assessment could be completed virtually and without supervision. In this task, listeners were asked to first read and comprehend sentences presented on a screen and to determine whether or not the sentence makes sense. Half of the sentences were absurd (e.g., “The fish drove a car”) and the other half were normal sentences (e.g., “The ball bounced away”). Each content word and any accompanying articles (e.g., “the ball” or “a car”) were presented sequentially on the screen each for 800 ms. Listeners were then asked to respond “yes” by pressing a button on the screen if the sentence made sense or “no” if the sentence was absurd. If listeners did not respond within 3,000 ms, the program advanced automatically. Participants were presented with 2–5 sentences per sequence. Listeners were then asked to recall either the first content word or the last content word from each sequence. They were not made aware beforehand whether they will be expected to recall the first or the last word, and thus must maintain both streams of information simultaneously. Using their keyboard, listeners typed their content word responses into a box on the screen and the number of correctly recalled words (not in correct serial order) out of the number of possible words was scored. Because participants were not supervised during this task, practice trials with feedback were provided. First, participants practiced only responding whether or not the sentence made sense. Next, they practiced recalling the first words of a two-sentence sequence, then the last words of a two-sentence sequence. Last, they practiced responding by recalling either the first or the last words of a two-sentence sequence before beginning the actual task. The percent correct of first or last words correctly recalled in any order (“*RST Percent Correct”*) reflects a participant’s complex working memory capacity.

#### Digit span forward and backward

The digit span task represents a traditional neuropsychological measure of a listener’s short-term memory (digit forward), such as the storage of a phone number (Jones and Macken, 2015), and simple working memory capacity (digit backward). Digit span backwards requires that the participant store and later invert the serial presentation of numerical information, similar to the storage and reprocessing of information during more demanding working memory tasks like the RST. Digit span forward and backward then may represent reduced processing demands compared to the RST (Daneman and Merikle, 1996) or different processes of working memory, with digit span forward and backward tapping into the simple rehearsal of visual stimuli during working memory and RST tapping into more complex rehearsal and reprocessing of visual information in the current study (Millman and Mattys, 2017). However, these complex working memory tasks correlate weakly with digit span backwards and the role of the digit span task as an assessment of working memory has been questioned (Hilbert et al., 2015). The digit span forward and backward task was chosen in addition to the RST to assess a range of memory capacities, from simple to complex, and their relationship to restoration of missing speech across participants. The current digit memory test was designed and revised by Turner and Ridsdale (2004). Participants were presented with a sequence of 2–9 digits and were afterwards asked to type them into the computer, either in the same order for digit span forward, or in reverse order for digit span backward. Each digit was presented on the screen for 1,000 ms. If participants typed in an incorrect response for both trials of a given sequence length, the task would end. Prior to administration of digit span forward and digit span backward, participants had two practice trials in which they received feedback for each task. Percentiles were calculated from norms and were based on the total number of correct trials for digit span forward and digit span backward together (“*DST Percentile”*) (Turner and Ridsdale, 2004).

#### Stroop task

The Stroop task measures a participant’s inhibitory control, or their ability to suppress task-irrelevant information. The ability to inhibit irrelevant verbal information, such as unlikely lexical candidates, may allow some listeners to restore more missing speech than others. In the Stroop task, participants named color words (*W*, 25 items) (e.g., “blue”), color hues of “XXXX” to eliminate any reading component (*C*, 25 items), and color words printed in an incongruent color hue (*CW*, 25 items) (e.g., “blue” written in green ink). For the incongruent trials, the participant was asked to name the color of the ink that the word is printed in, not the word itself. The task–naming color words–captures processing speed in milliseconds [“*Stroop Processing Speed (ms)*”], while the final task captures a participant’s interference score, with higher interference scores indicating reduced inhibitory control and poorer performance (Jensen, 1965). This assessment was based on the method developed by Golden (1976); however, rather than the number of items completed within a specified time limit, each participant completed the same number of items and correct/incorrect and reaction time for each item were captured. For each item, the participant pressed a key on their keyboard that corresponded with the first letter of the color (e.g., “b” for blue). Reminders for the keys were present on the screen. Interference was calculated as the ratio of the average time in milliseconds to correctly identify a CW trial divided by the average time taken to correctly identify a C trial (i.e., *CW/C*), a method common in neuropsychology literature [*“Stroop Interference (ms)*”] (Lansbergen et al., 2007; Scarpina and Tagini, 2017).

#### Flanker task

The Flanker task measures a participant’s response inhibition, or the ability to suppress responses that are irrelevant or inappropriate for a given task. The Flanker task requires participants to inhibit irrelevant non-verbal information, such as noise bursts, which may allow some listeners perform better on some perceptual restoration tasks than others. During this task, participants completed a computerized version of the Eriksen flanker task (Eriksen and Eriksen, 1974). During this task, participants were presented with five black arrows against a white background and were asked to press a key (“e” for left-facing arrows and “i” for right-facing arrows) to indicate the direction of the arrow in the middle. Participants were asked to respond as quickly and as accurately as possible. There was no time limit for responding on each trial. Half of the 90 items were congruent (e.g., >>>>> or <<<<<) and half were incongruent (e.g., >><>> or <<><<). The interstimulus interval was 750 ms. Before the scored trials, participants had eight practice trials in which they received feedback. Reaction time for congruent and incongruent items were captured as well as task accuracy. Interference was calculated by subtracting the mean reaction time for correct congruent items from the mean reaction time for correct incongruent items in milliseconds [“*Flanker Interference (ms)”*] (Sanders et al., 2018).

### Linguistic task

To assess listeners’ lexical access accuracy and lexical access speed, participants completed an automated virtual assessment in the visual modality, the Lexical Test for Advanced Learners of English (LexTALE; lexical knowledge and lexical access speed).

#### Lexical test for advanced learners of English

The English version of the LexTALE task (Lemhöfer and Broersma, 2012) estimates English vocabulary size (i.e., lexical knowledge) and the speed at which lexical decision-making occurs (i.e., lexical access speed). This measure was originally developed to assess lexical knowledge for intermediate to advanced learners of English as a second language. However, participants who speak English as their first language do not necessarily produce ceiling effects (Lorette and Dewaele, 2015) because factors such as age can influence lexical knowledge over time (Keuleers et al., 2015). Participants were presented with 60 items, 40 of which are real English words and 20 of which are orthographically permissible, pronounceable non-words. Participants were asked to press the “j” key if the word is a real word or the “k” key if it was a non-word and to respond as quickly and as accurately as possible. Reminders for the keys were present on the screen. Participants had 2,000 ms to respond before the program automatically advanced, scoring the missed item as incorrect. Participants did not receive practice trials or feedback prior to task administration. Lexical knowledge was the average of correct responses for real English words and non-words [*“LexTALE Non-word Accuracy (ms)”*] while lexical access speed was measured using the reaction time (“*LexTALE Word RT”*) of correctly identified real words.

### Screening task

Participants were asked to wear headphones and to set the volume on their computer to a “loud, but not uncomfortable” level while listening to a recorded excerpt from the Discourse Comprehension Test (Brookshire and Nicholas, 1984). Listeners were also asked to complete a headphone screening procedure to ensure headphone use [see Woods et al. (2017) for more detail]. Briefly, the headphone test required the listener to listen to three tones and pick the softest one out of three correctly at least 4/6 times. Over a loudspeaker setup (e.g., laptop), one of the three tone presentations suffers from destructive interference resulting from two tones presented out of phase at each loudspeaker, making it difficult to differentiate from the tone that is 6 dB below the standard tone. With headphones, the phase differences do not result in destructive interference, making one of the three tones easier to pick out as the softest. Failing the headphone screening twice resulted in exclusion.

### Interrupted speech task

Participants listened to and practiced typing in uninterrupted sentences, followed by those same sentences interrupted by both silent gaps and noise bursts. Feedback was not provided. Participants then listened to the experimental interrupted stimuli. The order of the silent gap sentences and the noise burst sentences were blocked and counterbalanced to prevent order effects. Within each (silent gap or noise burst) block, sentences were not blocked by sentence type and were presented in a random order. After one RSPIN or PRESTO sentence was presented, listeners were asked to type in what they heard into a box on the computer screen. The number of keywords correctly identified was scored using Autoscore (Borrie et al., 2019). Autoscore is an open-source tool for scoring listener transcripts, where the researcher specifies the scoring rule and under which circumstances that rule should be applied. Strict criterion were applied in this experiment. Only the double-letter rule was applied, which scores a word as correct if a double letter is omitted within a word (e.g., “atack” is considered correct for “attack”). Additionally, a custom acceptable spelling list was created that included common misspellings of all keywords in the RSPIN and PRESTO sentences including the following: single letter transpositions within a single word during typing, inclusion/omission of an apostrophe for keywords with a contraction, and any entry of a double space (e.g., spacebar was accidentally hit twice). Traditionally, only the last word of the RSPIN is scored; however, we were interested in how participants restored speech across the entire interrupted sentence, not just the word in the final position. Therefore, content words across the entire sentence were scored using the same method and number of keywords as Jenstad and Souza (2007).

### Statistical approach

All data were analyzed using the open source RStudio statistical program version 4.0.5 (R Core Team, 2013), using the *tidyverse* library (Wickham et al., 2019) including the library *dplyr* for data manipulation (Wickham et al., 2022) prior to statistical analysis. The library *ggplot2* was also used for data visualization and figure preparation (Wickham, 2016). For the analysis of variance, the library *rstatix* was utilized (Kassambara, 2021). For the linear models, the libraries *MASS* and *lmtest* were used to assess homoscedasticity and the distribution of residuals (Venables and Ripley, 2002; Zeileis and Hothorn, 2002). First, outliers in the data were identified and adjusted, followed by descriptive analysis for participant data, cognitive and linguistic measures, and interrupted speech conditions. Next, an analysis of variance (ANOVA) tested for significant differences between the six sentence conditions and Pearson correlations between cognitive and linguistic variables and interrupted sentence conditions were determined. Last, a set of linear regression analyses was performed using cognitive and linguistic variables as predictors for the six sentence conditions.

## Results

Prior to analysis, outliers were identified and adjusted, and a fence was determined. All values within any single measure that were outside three times the interquartile range (IQR) were identified as outliers and were adjusted to the nearest fence boundary (i.e., the first or third quartile) to minimize regression toward the mean. Three times the IQR was chosen as a conservative fence in order to avoid unnecessary adjustment given the unsupervised, online nature of the data collected. In total, nine of 1,071 observations across the seventeen reported measures fell outside of the IQR fence and were adjusted. Of the nine adjusted observations, six occurred in the linear models that follow. Descriptive statistics for the 63 participants in this study are presented in Table 1 and results for cognitive and linguistic measures and interrupted speech conditions are available in Table 2. After addressing outliers, measures were normally distributed with skewness and kurtosis under accepted values (Kline, 2015). Participants in this sample performed slightly better but within one standard deviation on the RST compared to existing data (Friedman and Miyake, 2005; Füllgrabe et al., 2015), performed above average on the digit span task with an average percentile score of 72.6 (Turner and Ridsdale, 2004), were consistent with existing Stroop data with regard to reaction time but slightly better with regard to interference scores (Langenecker et al., 2004; Van der Elst et al., 2006), Flanker interference scores were within one standard deviation of existing data (Paap et al., 2020), and participants were highly consistent with published data for English monolinguals on the LexTALE task (Dijkgraaf et al., 2016).

**TABLE 1:** Descriptive statistics for participants.

Measures	Mean (SD)	Range	Skew	Kurtosis
Age	42.0 (12.0)	[22,63]	–0.04	–1.2
Education	15.4 (2.8)	[8,24]	0.27	0.79
SSQ–speech	8.4 (1.4)	[10,3]	–1.25	2.11
SSQ–spatial	7.7 (1.5)	[10,4]	–0.4	–0.48
SSQ–qualities	8.6 (1.2)	[10,5]	–1	0.39

**TABLE 2:** Descriptive data for experimental tasks.

	Measures	Mean (SD)	Range	Skew	Kurtosis
Cognitive measures	RST percent correct	73.5 (14.3)	[96.7,34]	–1.31	1.66
	DST percentile	72.6 (28.6)	[99.9, 0.8]	–1.22	0.42
	Stroop interference (ms)	1.35 (0.26)	[2.29, 0.95]	1.07	1.33
	Stroop processing speed (ms)	804.9 (156.9)	[1,337,472]	0.84	1.41
	Flanker interference (ms)	41 (23.7)	[100, −12.5]	0.52	0.21
Lexical tasks	LexTALE percent correct	88.7 (9.4)	[100, 66.3]	–1.13	0.45
	LexTALE word RT (ms)	754.3 (125.5)	[1,110,476]	0.52	0.4
Interrupted speech conditions	RSPIN high silent	48.3 (8.7)	[64,26]	–0.55	–0.32
	RSPIN high noise	60.1 (9.1)	[77,31]	–0.97	1.28
	RSPIN low silent	42.4 (5.9)	[57,30]	0.2	0.01
	RSPIN low noise	50.4 (7.7)	[65,25]	–0.66	0.84
	PRESTO silent	27.6 (8.3)	[47,3]	–0.03	0.46
	PRESTO noise	43.1 (8.7)	[57,21]	–0.3	–0.54

RSPIN, revised speech in noise test; PRESTO, perceptually robust English sentence test open-set; High refers to high context sentences; Low refers to low context sentences; Silent refers to sentences interrupted by a silent gap; and Noise refers to sentences interrupted by a noise burst.

### Perceptual restoration differences across experimental conditions

Number of keywords correctly identified across the six sentence conditions were analyzed using an analysis of variance (ANOVA) with the sentence conditions as factor levels and the percent of keywords correctly identified as the dependent variable. The normality assumption was checked and met using quantile-quantile (Q-Q) plots rather than a Shapiro-Wilk test, as the sample size is greater than 50 participants (D’Agostino, 1971). Levene’s test for the homogeneity of variances assumption necessary for the ANOVA was significant, indicating the variances for the six sentence conditions were not equal *F*_(5,372)_ = 2.45, *p* = 0.03 (Levene, 1960). To account for this violation, a Welch one-way test was used which does not require homogeneity of variance (Moder, 2007).

**FIGURE 1:**
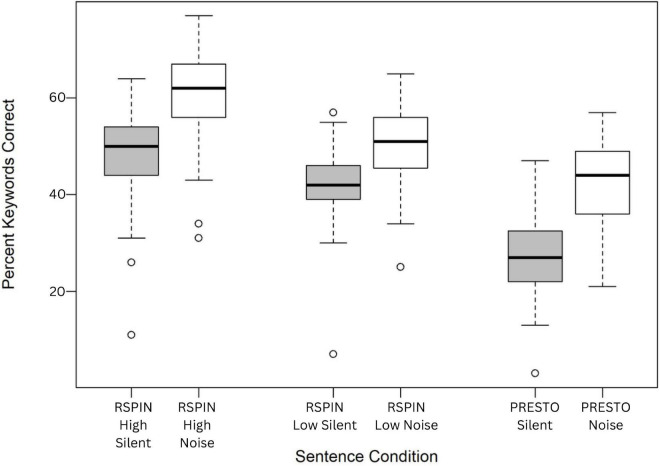
Box plot representing perceptual restoration, or the percent of keywords correctly identified for the six sentence conditions.

The perceptual restoration of missing speech information differed by sentence condition (Figure 1), *F*_(5,372)_ = 99.7, *p* = < 0.001. *Post-hoc* pairwise *t*-tests with no assumption of equal variances using a Benjamini-Hochberg correction for multiple comparisons revealed that all pairwise differences between the six conditions were statistically significant (*p* < 0.05) and different from one another, except PRESTO Noise and RSPIN Low Silent conditions (*p* = 0.58) and RSPIN High Silent and RSPIN Low Noise conditions (*p* = 0.18) (Benjamini and Hochberg, 1995).

### Relationships between perceptual restoration and higher-order, cognitive and linguistic variables

**FIGURE 2:**
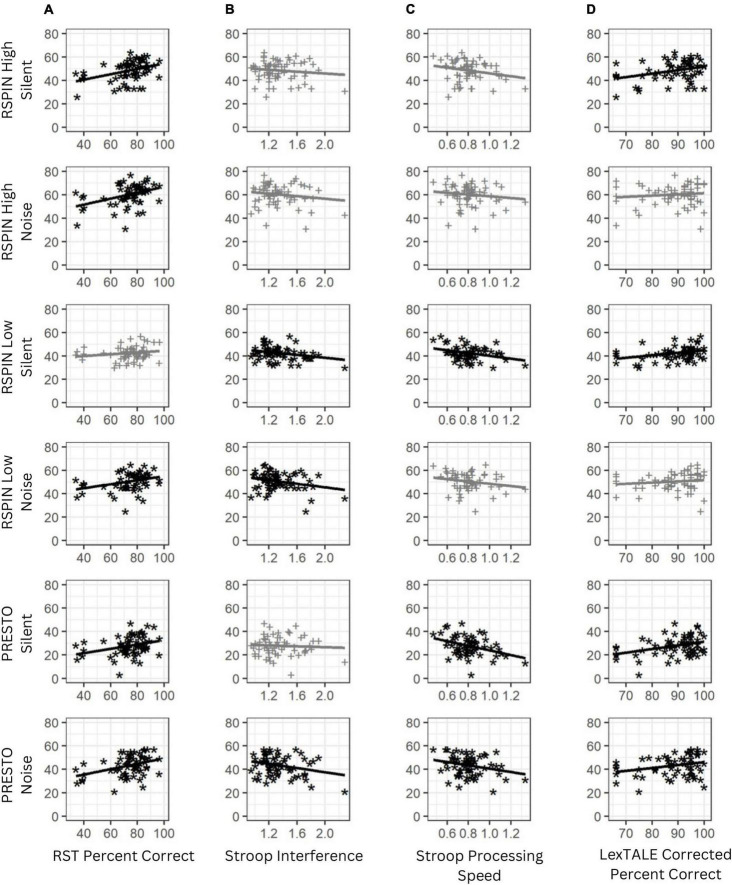
Scatter plots of perceptual restoration scores for interrupted speech conditions with working memory capacity measured using the reading span test (RST) **(A)**, inhibitory control measured using the Stroop task **(B)**, processing speed measured using Stroop reaction time **(C)**, and lexical accuracy measured using the LexTALE Percent Correct score **(D)**. Higher scores on RST Percent Correct and LexTALE Percent Correct indicate better performance, while lower scores on Stroop Interference and Stroop Processing Speed indicate better performance. Scatter plots in black with asterisk symbols are statistically significant without correction for multiple comparisons (*p* < 0.05) and scatter plots in gray with plus symbols are not statistically significant. Refer to Table 3 for correlation strength and statistical significance for each of these measures.

Correlations between the six sentence conditions and cognitive and linguistic variables are presented in Table 3 and Figure 2 (note that *p*-values have not been adjusted for multiple comparisons). Complex working memory capacity measured with the Reading Span Task was moderately correlated with simple working memory measured using the digit span task, a traditional measure working memory thought to be less taxing than complex working memory tasks (*r* = 0.28, *p* = 0.02). This is consistent with previous research (Daneman and Merikle, 1996) and with similar construct validity between complex working memory, or tasks requiring substantial information storage and reprocessing, and simple working memory, or tasks requiring more straightforward repetition or reversal of information (Lehto, 1996), though the digit span task was not correlated with interrupted speech performance and may not necessarily represent working memory performance (Jones and Macken, 2015). Furthermore, working memory capacity had a moderate, negative correlation with inhibitory control measured using the Flanker task (*r* = –0.28, *p* = 0.02), which was the only significant correlation with the Flanker task across all measures, making it a weak predictor overall. Lexical processing speed recorded using the LexTALE word reaction time in milliseconds was positively and significantly correlated with inhibitory control measured using the Stroop Interference score (*r* = 0.29, *p* = 0.02) and processing speed measured using the Stroop word-only item reaction time, or processing speed (*r* = 0.29, *p* = 0.02). This result is consistent with both processing speed and the calculation of inhibitory control both relying on reaction time. Working memory measured using the Reading Span Task significantly and positively correlated with five of the six sentence conditions. Lexical knowledge measured using the LexTALE percent correct score correlated with four of the six sentence conditions. Both inhibitory control measured using the Stroop interference score and processing speed measured using the Stroop word-only item reaction time correlated with three of the six sentence conditions. After correcting for multiple comparisons using the Bonferroni method for 24 comparisons (six conditions times four measures of interest), reducing the α level to 0.00208, only RST was significantly correlated with RSPIN High Silent (*r* = 0.38, *p* = 0.002), RSPIN High Noise (*r* = 0.40, *p* = 0.001), and PRESTO Noise (*r* = 0.38, *p* = 0.002).

**TABLE 3:** Pearson correlation coefficients across cognitive, linguistic, and interrupted speech measures.

Measures		1	2	3	4	5	6	7	8	9	10	11	12
**Cognitive measures**													
1	RST percent correct	1											
2	DST percentile	**0.28 (0.02)**	1										
3	Stroop interference (ms)	0.005 (0.96)	0.003 (0.98)	1									
4	Stroop processing speed (ms)	−0.15 (0.25)	0.07 (0.55)	−0.07 (0.60)	1								
5	Flanker interference (ms)	**−0.28 (0.025)**	−0.12 (0.38)	0.08 (0.51)	0.13 (0.30)	1							
**Linguistic measures**													
6	LexTALE percent correct	0.04 (0.74)	−0.10 (0.43)	−0.19 (0.12)	−0.13 (0.29)	−0.15 (0.21)	1						
7	LexTALE word RT (ms)	−0.22 (0.08)	−0.02 (0.86)	**0.29 (0.02)**	**0.29 (0.02)**	0.08 (0.51)	−0.18 (0.17)	1					
**Interrupted speech measures**													
8	RSPIN high silent	**0.38 (0.002)**	−0.03 (0.82)	−0.12 (0.36)	−0.22 (0.08)	−0.11 (0.40)	**0.32 (0.009)**	−0.12 (0.37)	1				
9	RSPIN high noise	**0.40 (0.001)**	−0.05 (0.68)	−0.16 (0.22)	−0.13 (0.32)	−0.01 (0.94)	0.10 (0.39)	−0.18 (0.15)	**0.63 (<0.001)**	1			
10	RSPIN low silent	0.18 (0.16)	−0.1 (0.43)	**−0.27 (0.03)**	**−0.32 (0.01)**	0.001 (0.99)	**0.34 (0.006)**	−0.13 (0.31)	**0.73 (<0.001)**	**0.47 (<0.001)**	1		
11	RSPIN low noise	**0.32 (0.01)**	−0.04 (0.74)	**−0.27 (0.03)**	−0.21 (0.11)	−0.04 (0.74)	0.13 (0.30)	−0.22 (0.08)	**0.62 (<0.001)**	**0.83 (<0.001)**	**0.55 (<0.001)**	1	
12	PRESTO silent	**0.32 (0.01)**	−0.07 (0.59)	−0.06 (0.64)	**−0.38 (0.002)**	−0.08 (0.56)	**0.35 (0.004)**	−0.21 (0.09)	**0.80 (<0.001)**	**0.48 (<0.001)**	**0.80 (<0.001)**	**0.57 (<0.001)**	1
13	PRESTO noise	**0.38 (0.002)**	0.006 (0.96)	**−0.27 (0.03)**	**−0.26 (0.04)**	−0.05 (0.67)	**0.26 (0.03)**	**−0.27 (0.03)**	**0.78 (<0.001)**	**0.79 (<0.0001)**	**0.63 (<0.001)**	**0.79 (<0.001)**	**0.67 (<0.001)**

Correlation strength is followed by statistical significance, and bolded cells are statistically significant without correction for multiple comparisons (*p* < 0.05).

Some measures had few or no correlations with the interrupted sentence conditions. For example, lexical access speed measured using the LexTALE correctly identified word reaction time in milliseconds correlated only with the PRESTO noise-burst interrupted sentence condition. Simple working memory measured using the digit span task and inhibitory control measured using the Flanker task did not correlate with any of the sentence conditions. These latter three variables were considered poor predictors and were excluded from further analysis. Age was significantly correlated with only the PRESTO silent gap interrupted sentence condition (*r* = –0.4, *p* = 0.001) and correlated with only the Stroop processing speed cognitive measure (*r* = 0.35, *p* = 0.004). Age was not significantly correlated with perceptual restoration performance or performance on the cognitive and linguistic measures overall and was excluded from further analysis.

Linear regression analysis was performed using normalized predictors and word recognition percent correct outcome data. Separate models were conducted for each sentence condition. Predictors for the models for the sentence conditions were selected using an *a priori*, hypothesis-driven approach. This approach was informed by the results of Table 3 to minimize Pearson correlation coefficients between predictors during linear model design (Bursac et al., 2008; Hosmer et al., 2013). Last, *a priori* model design was checked against a quantitative approach to minimize the number of predictors while maximizing numerical stability and ease of interpretation [i.e., purposeful selection (Zhang, 2016)]. This approach removes predictors, one by one, from a full, saturated model when their *p*-values are less than 0.25, unless they are assumed to be related to the hypothesis (Mickey and Greenland, 1989). During this process, predictors such as age, education, the SSQ, DST, and Flanker were not significantly associated with the six interrupted speech conditions and were systematically removed from the model. This method then creates a new, smaller model which can be compared to the saturated model to ensure that the change in coefficients (Δβ) is not greater than 20%, which would indicate that these predictors should be added back into the model given their strong adjustment effect. Last, potential interactions among remaining predictors are checked one-by-one and removed if non-significant before goodness of fit (GOF) is checked visually using plots of residual values versus fitted values and Q-Q plots. This purposeful selection approach was completed for each sentence condition using the percent of words correctly identified as outcome. This process resulted in an overall standard model with the same four predictors used in each model for ease of interpretation: RST Percent Correct, LexTALE Percent Correct, Stroop Interference, and Stroop Processing Speed. Multicollinearity was deemed acceptable among these predictors with the highest variance inflation factor value being 1.06, very close to the minimum value of 1 and well below 2.5, which may indicate multicollinearity, or 10, which is problematic (Mansfield and Helms, 1982; Vittinghoff et al., 2006; Thompson et al., 2017; Johnston et al., 2018). All six models meet the assumption of homoscedasticity necessary for linear model design using a studentized Breusch-Pagan test (Breusch and Pagan, 1979). Three (RSPIN Low Silent, PRESTO Silent, and PRESTO Noise) models met the assumption that the residuals are normally distributed using a Wilk-Shapiro test of normality, while the remaining three models (RSPIN High Silent, RSPIN High Noise, RSPIN Low Noise) have a non-normal distribution of residuals and should be interpreted with caution.

The overall linear models for all six conditions were statistically significant (*p* < 0.05). All six models are reported without correction for multiple comparisons (see Table 4) as there is not a strong consensus regarding correction when considering multiple separate models. However, a Bonferroni correction for six comparisons reduces the α level to 0.008 and five of the six models remain significant, with only RSPIN High Noise losing significance (*p* = 0.01) Adjusted R^2^ values ranged from 13.6 for RSPIN High Noise to 25.5 for PRESTO Silent. Working memory capacity measured using the RST was a significant predictor in five of the six models: RSPIN High Silent (β = 3.01, *p* = 0.004), RSPIN High Noise (β = 3.51, *p* = 0.002), RSPIN Low Noise (β = 2.28, *p* = 0.015), PRESTO Silent (β = 2.16, *p* = 0.02), and PRESTO Noise (β = 2.98, *p* = 0.003). Lexical knowledge measured using the LexTALE task was a significant predictor for restoring missing speech for silent conditions, but not noise conditions: RSPIN High Silent (β = 2.40, *p* = 0.022), RSPIN Low Silent (β = 1.47, *p* = 0.04), and PRESTO Silent (β = 2.48, *p* = 0.01). On the other hand, inhibitory control measured using the Stroop task was a significant predictor for the majority of noise burst conditions and one silent gap condition: RSPIN Low Silent (β = –1.45, *p* = 0.04), RSPIN Low Noise (β = –2.09, *p* = 0.027), and PRESTO Noise (β = –2.17, *p* = 0.03). Finally, processing speed significantly predicted perceptual restoration ability in only two of the six models, but in most of the silent gap conditions: RSPIN Low Silent (β = –1.67, *p* = 0.02), and PRESTO Silent (β = –2.48, *p* = 0.01).

**TABLE 4:** Linear regression analysis models predicting perceptual restoration by sentence condition.

Condition	Predictors	R^2^ (%)/Adjusted R^2^ (%)	*F* (*p*-value)	β	Confidence intervals	*T*-score (*p*-value)
RSPIN high silent		**25.8/20.7**	**5.05 (0.001)**			
	*RST percent correct*			**3.01**	**1.01–5.01**	**3.02 (0.004)**
	Stroop interference			−0.66	−2.69–1.36	−0.66 (0.51)
	Stroop processing speed			−1.21	−3.23–0.81	−1.2 (0.24)
	*LexTALE percent correct*			**2.40**	**0.36–4.44**	**2.36 (0.022)**
RSPIN high noise		**19.2/13.6**	**3.45 (0.01)**			
	*RST percent correct*			**3.51**	**1.33–5.69**	**3.22 (0.002)**
	Stroop interference			−1.42	−3.63–0.79	−1.28 (0.2)
	Stroop processing speed			−0.69	−2.90–1.51	−0.63 (0.53)
	LexTALE percent correct			0.47	−1.75–2.69	0.42 (0.67)
RSPIN low silent		**26.3/21.3**	**5.18 (0.001)**			
	RST percent correct			0.76	−0.59–2.12	1.13 (0.26)
	*Stroop interference*			**−1.45**	**−2.82–−0.08**	**−2.12 (0.04)**
	*Stroop processing speed*			**−1.67**	**−3.04–−0.30**	**−2.44 (0.02)**
	*LexTALE percent correct*			**1.47**	**0.09–2.85**	**2.14 (0.04)**
RSPIN low noise		**21/15.5**	**3.84 (0.007)**			
	*RST percent correct*			**2.28**	**0.46–4.1**	**2.51 (0.015)**
	*Stroop interference*			**−2.09**	**−3.93–−0.25**	**−2.27 (0.027)**
	Stroop processing speed			−1.35	−3.19–0.5	−1.46 (0.15)
	LexTALE percent correct			0.34	−1.52–2.19	0.36 (0.72)
PRESTO silent		**30.3/25.5**	**6.3 (0.0002)**			
	*RST percent correct*			**2.16**	**0.33–4.00**	**2.36 (0.02)**
	Stroop interference			−0.18	−2.04–1.68	−0.19 (0.84)
	*Stroop processing speed*			**−2.48**	**−4.34–−0.62**	**−2.67 (0.01)**
	*LexTALE percent correct*			**2.48**	**0.61–4.35**	**2.65 (0.01)**
PRESTO noise		**29.8/24.9**	**6.14 (0.0003)**			
	*RST percent correct*			**2.98**	**1.05–4.92**	**3.09 (0.003)**
	*Stroop interference*			**−2.17**	**−4.13–−0.21**	**−2.22 (0.03)**
	Stroop processing speed			−1.74	−3.69–0.22	−1.78 (0.08)
	LexTALE percent correct			1.55	−0.42–3.52	1.58 (0.12)

Significant predictor titles are italicized with bolded statistics (*p* < 0.05).

## Discussion

The current study was designed to investigate the role of higher-order cognitive and linguistic abilities, such as working memory capacity, lexical knowledge (i.e., vocabulary), lexical access speed, inhibitory control, and processing speed during the perceptual restoration of missing speech information in adults. Of the measures tested, working memory capacity was the most predictive cognitive ability and lexical knowledge was the most predictive linguistic ability during the restoration of missing speech information. The strength of contribution depended on the type of interruption and on sentence material. In the silent gap conditions, a larger set of cognitive and linguistic abilities predicted the restoration of missing speech information. In the noise burst conditions, only working memory capacity and inhibitory control predicted perceptual restoration ability. For high context sentences, working memory capacity and linguistic knowledge predicted the restoration of missing speech, whereas, in the low context and everyday speech conditions, a larger set of cognitive and linguistic abilities predicted perceptual restoration.

### Perceptual restoration of missing speech by interruption type and sentence type

In line with previous research, listeners restored more missing speech when sentences were interrupted with noise bursts rather than silent gaps, regardless of sentence type (Miller and Licklider, 1950; Warren, 1970; Powers and Wilcox, 1977; Bashford et al., 1992; Bologna et al., 2019). This result is thought to occur because of gestalt properties of perceptual organization supporting the percept of continuous speech occurring behind the noise bursts, as long as the noise burst itself would be considered an effective masker of the target signal (Warren and Obusek, 1971; Bashford et al., 1992). The noise bursts may also mask the accidental perception of word boundary that might occur during silent gap interrupted sentences. This occurs when a word is interrupted by a silent gap and that same silent gap is misinterpreted as the end of a word, resulting in the percept of a non-word. However, the noise burst may override this challenging effect by creating illusory continuity and improving degraded speech recognition (Clarke et al., 2016).

Listeners were also able to benefit from sentence context, attaining better scores for RSPIN High sentences when compared to RSPIN Low sentences, a result that is in line with existing perceptual restoration literature (Bashford and Warren, 1987; Bashford et al., 1992; Kidd and Humes, 2012). However, the benefit of context may be limited by the constraints of the RSPIN sentences themselves. Originally, the RSPIN sentences were designed so that only the last word of each sentence would be scored as either correct or incorrect (Bilger et al., 1984). In our data the entire sentence was periodically interrupted and participant performance was scored across all key words in the sentence. Scoring only the last word may reduce individual differences in the ability to compensate across an entire sentence, because sentence context effects take place across an entire sentence (Stanovich and West, 1983; Kutas et al., 2019), the effect builds over time when sentences are predictable (Brothers and Kuperberg, 2021), and high predictability increases the benefit from glimpses of target speech across an entire sentence (Schoof and Rosen, 2015). However, it should be noted that the RSPIN High Silent, RSPIN High Noise, and RSPIN Low Noise sentences had a non-normal distribution of residuals in the current data set and thus these results should be interpreted with caution and RSPIN High Noise did not survive a Bonferroni correction for multiple comparisons.

The highest variability across listeners and poorest performance occurred for PRESTO sentences. This increase in variability and decrease in performance may have occurred for several reasons. First, the PRESTO sentences were designed to incorporate multiple factors during speech recognition: talker characteristics, dialect, and the role of higher order processes. The PRESTO sentences also vary in length and syntactic complexity. Taken together, these factors make PRESTO sentences less constrained than the RSPIN sentences and, thus, more representative of everyday speech (Cole et al., 2010). Second, the PRESTO sentences used here contain 455 unique words, which exceeds both the RSPIN High (421 words) and RSPIN Low (218 words) conditions. Therefore, the variability in the results may follow simply from the increased variability in the number of unique words in the PRESTO sentence set. Third, many of the key words in the PRESTO sentence set are longer and contain additional syllables (average of 7.96 syllables per sentence for PRESTO sentences compared to 6.14 syllables per sentence for the RSPIN High and 6.29 for the RSPIN Low context sentence sets). While the silent gap and noise burst interruptions in the current experiment were designed to be shorter than the average syllabus nuclei duration in American English (Peterson and Lehiste, 1960), minimizing the obliteration of syllables entirely (Miller and Licklider, 1950), additional syllables in a key word may provide listeners with multiple glimpses at one word, which may improve or support perceptual restoration ability. This wider range of syllabic structure may contribute to the increased variability in the PRESTO sentences compared to the RSPIN sentences.

### The role of working memory capacity in perceptual restoration

Working memory capacity measured using the Reading Span Task was significantly correlated with or acted as a significant predictor for interrupted speech recognition in five of the six sentence conditions. The significance of working memory capacity is in line with some previous literature for noise burst interrupted sentences using low-context, QuickSIN stimuli and a similar interruption paradigm, indicating the importance of working memory capacity for noise burst sentences (Millman and Mattys, 2017; Nagaraj and Magimairaj, 2017). However, previous literature has also found that working memory capacity does not play a role during perceptual restoration of PRESTO sentences using a very similar interruption process (Bologna et al., 2018). Bologna and colleagues used a zero signal-to-noise ratio (SNR) for the noise burst stimuli so that the speech was the same overall intensity as the noise bursts. This design may reduce the percept of speech continuity behind the noise burst, which may impede or interfere with the reprocessing role of working memory capacity during perceptual restoration, making the task more difficult than a noise burst condition with a negative SNR as in the current study. This would fall in line with existing literature that indicates few significant correlations between working memory capacity and silent gap interrupted sentences (Nagaraj and Knapp, 2015; Shafiro et al., 2015; Jaekel et al., 2018). A unique aspect of the current study is the wider range of participant age compared to most existing data, which tested only younger participants (Nagaraj and Knapp, 2015; Nagaraj and Magimairaj, 2017) or utilized group comparisons between older and younger adults, largely missing middle-aged listeners (Shafiro et al., 2015; Millman and Mattys, 2017; Bologna et al., 2018; Jaekel et al., 2018). Given the changing role that working memory capacity plays with increasing age (Wingfield et al., 1988), its effect on language comprehension (Caplan and Waters, 2005), and its possible task dependent nature (Turner and Engle, 1989), this may explain why the current data set found significant working memory capacity correlations for the majority of difficult silent gap conditions.

### Lexical knowledge, lexical access speed, and perceptual restoration

The current data add to the evidence that lexical knowledge is important during perceptual restoration (Benard et al., 2014; Nagaraj and Magimairaj, 2017; Bologna et al., 2018; Jaekel et al., 2018). Under the ELU model, lexical knowledge is thought to support explicit working memory reprocessing within the episodic buffer (Rönnberg et al., 2013). This explicit reprocessing identifies likely and unlikely lexical candidates for the missing speech segments and attempts to reconcile segments into a cohesive, logical whole across the entire utterance (Bashford et al., 1992; Zhang and Samuel, 2018). In this way, the most lexically and contextually appropriate candidate can then be chosen by comparing options at the sentence level rather than just the gap level, thereby improving perceptual restoration across the entire utterance (Bashford and Warren, 1987).

For the silent gap interrupted conditions where lexical knowledge was strongly predictive, it is feasible that for listeners with greater lexical knowledge that a larger set of possible lexical candidates might be identified in the silent gap conditions than for listeners with poorer vocabularies. For the noise burst sentences where lexical knowledge was less predictive, it is possible that the noise burst itself may create enough illusory perceptual continuity that the correct lexical candidate can be more easily identified for all listeners, regardless of vocabulary size (Bashford et al., 1996). Alternatively, listeners with greater lexical knowledge may be less susceptible to misidentification of word boundaries in the silent gap conditions, making them better able to activate appropriate lexical candidates despite incomplete lexical neighborhood activation (Clarke et al., 2016). This alternative hypothesis follows the Neighborhood Activation Model, which suggests that listeners with greater lexical knowledge, even without priming, are better able to activate lexical neighborhoods with incomplete information, and that this effect is only detectable in the silent gap interrupted conditions because the noise burst sentences facilitate enough lexical neighborhood activation for all listeners (Luce and Pisoni, 1998; Luce et al., 2000).

To date, no known data have been reported on the relationship between lexical access speed, or the rate at which lexical candidates are identified and selected, and perceptual restoration. The current data do not support a significant role of lexical access speed. The lack of results for lexical access speed may stem from the LexTALE task itself, which was not designed to assess lexical access speed but rather to assess English language proficiency for English second language learners (Lemhöfer and Broersma, 2012), though it does have predictive value as a rapid task of proficiency assessment in English first language learners (Lorette and Dewaele, 2015). Although a computerized assessment does allow for the capture of reaction time for real words, non-words, and correct and incorrect items, the upper time limit of 2,000 ms may artificially limit lexical access speed. Future studies of perceptual restoration and lexical access speed should include measures designed to capture this time-sensitive measure.

### Inhibitory control, processing speed, and perceptual restoration

Inhibitory control was significantly correlated with and acted as a significant model predictor for three of the six sentence conditions: RSPIN Low Silent, RSPIN Low Noise, and PRESTO Noise. These results contrast those by Bologna et al. (2018), who found that inhibitory control did not significantly improve model fit for perceptual restoration in either silent gap or noise burst sentences. One possibility for the discrepancy between the current data and the results from Bologna et al. (2018) is the administration of the Stroop task. In the current data, the Stroop task was administered using an online platform, and listeners were asked to press a corresponding color key (e.g., “g” for green) when responding and do so as rapidly as possible. Remembering key location, key correspondence, and the motor control necessary to complete the task may have engaged working memory beyond what occurs during the process of responding verbally in the traditional administration of the Stroop task. Our Stoop task was correlated with and a significant predictor for most of the noise burst sentence conditions. This may indicate that inhibitory control plays an active role in inhibiting the irrelevant noise bursts when reprocessing speech fragments during perceptual restoration, and that listeners who are better able to inhibit the noise bursts are better able to focus on cognitive tasks that restore missing speech. However, RSPIN High Noise and RSPIN Low Noise conditions had a non-normal residuals distribution and this result should be interpreted with caution and RSPIN High Noise did not survive a Bonferroni correction for multiple comparisons.

Potential limitations of this experiment include the inability to measure audiometric thresholds from participants in this study, relying on self-report measures of “normal hearing.” Because auditory thresholds decline with increasing age (Gates and Mills, 2005; Huang and Tang, 2010) it is possible that hearing acuity may have had an unmeasured impact on the results, despite the lack of significant correlations with both age and SSQ on cognitive/linguistic data and restoration of missing speech. Next, it should be noted that the cognitive/linguistic measures in this study were all in the visual modality while the outcome measures of interest were in the auditory modality. While many of the cognitive and linguistic measures included in this study are often thought of as domain-general (i.e., they are not modality specific), there is evidence that modality differences may affect how signals are processed cortically (Salthouse and Meinz, 1995; Crottaz-Herbette et al., 2004; Roberts and Hall, 2008) which may influence these results. Furthermore, the scoring method chosen for cognitive/linguistic measures can often yield different results and the results from this study should be compared only to other measures administered in a similar fashion (Knight and Heinrich, 2017). Last, because the noise burst conditions are generally perceived as being less difficult than the silent gap conditions and the sentence types (e.g., RSPIN High, RSPIN Low, and PRESTO) differ from one another with regard to sentence and word length, these conditions may differ from one another with regard to overall task difficulty which can affect overall response accuracy (Robinson, 2001).

In the current experiment, processing speed, or the rate at which cognitive tasks are completed, was significantly correlated with RSPIN Low Silent, PRESTO Silent, and PRESTO Noise conditions and acted as a significant predictor in the RSPIN Low Silent and PRESTO Silent conditions. These results are similar to those found by Bologna et al. (2018) who found that interrupted key word recognition improved with faster processing speed when measured using the connections line making test (Salthouse, 2000). Given that processing speed was significant in two of three silent gap conditions, it is possible that these conditions are more difficult compared to the noise burst conditions and listeners who are able to reprocess and reanalyze the information more rapidly might be better able to restore missing speech information.

## Conclusion

In this study, we hypothesized that higher-order cognitive and linguistic abilities would facilitate the restoration of missing speech information using the ELU model framework (Rönnberg et al., 2013). The interrupted speech paradigm was utilized to explore this hypothesis, which in this case removed 50% of the speech signal in order to encourage participants to explicitly reprocess and reanalyze the incomplete speech signal. We predicted that listeners with stronger cognitive and linguistic abilities measured using validated cognitive measures would restore more missing speech information than those with weaker cognitive and linguistic abilities. Working memory capacity and lexical knowledge (i.e., vocabulary) played the most consistent and unique role in perceptual restoration across the sentence conditions, followed by inhibitory control and processing speed. In general, silent gap conditions appeared to be related to a broader range of cognitive and linguistic abilities whereas noise burst conditions were predicted by and correlated with working memory capacity and inhibitory control. Furthermore, sentences that had limited context cues and lacked predictability or were more like those encountered in everyday listening were significantly correlated with and predicted by a wider range of cognitive and linguistic abilities than those that contained additional context cues and had higher levels of predictability. The differences between silent gap and noise burst conditions as well as the context, predictability, and everyday speech conditions may be related to task-dependent difficulties that recruit different constellations of cognitive and linguistic abilities to facilitate the restoration of missing speech information. In sum, perceptual restoration of speech is a complex process that relies on an individual’s ability to store and reprocess, to identify potential lexical candidates, to inhibit irrelevant information, to contextually consider several options simultaneously, and to complete these cognitive tasks rapidly, and listeners vary considerably in these abilities (Carroll and Maxwell, 1979; Reuter-Lorenz et al., 2000; Cabeza et al., 2002; George et al., 2007; Rudner et al., 2008; Boogert et al., 2018).

## Data availability statement

The raw data supporting the conclusions of this article will be made available by the authors, without undue reservation.

## Ethics statement

The studies involving human participants were reviewed and approved by Northwestern University Institutional Review Board. The patients/participants provided their written informed consent to participate in this study.

## Author contributions

Both authors contributed to study design, data collection, management, analysis, and manuscript preparation and approved the submitted version.
